# Racial inequalities in preterm birth in Central-West Brazil, 2015–2024: a population-based cross-sectional study

**DOI:** 10.11606/s1518-8787.2026060007625

**Published:** 2026-09-17

**Authors:** Guilherme Coelho, Charles M’poca Charles, Rodolfo de Carvalho Pacagnella, Clarimar José Coelho, Danielle Satie Kassada

**Affiliations:** I Universidade Estadual de Campinas Faculdade de Ciências Médicas Programa de Pós-Graduação em Tocoginecologia Campinas SP Brasil Universidade Estadual de Campinas. Faculdade de Ciências Médicas. Programa de Pós-Graduação em Tocoginecologia. Campinas, SP, Brasil; II Universidade Estadual de Campinas Faculdade de Ciências Médicas Departamento de Tocoginecologia Campinas SP Brasil Universidade Estadual de Campinas. Faculdade de Ciências Médicas. Departamento de Tocoginecologia. Campinas, SP, Brasil; III Pontifícia Universidade Católica de Goiás Escola Politécnica e de Artes Departamento de Computação Goiânia GO Brasil Pontifícia Universidade Católica de Goiás. Escola Politécnica e de Artes. Departamento de Computação. Goiânia, GO, Brasil; IV Universidade Estadual de Campinas Faculdade de Enfermagem Departamento de Saúde Coletiva Campinas SP Brasil Universidade Estadual de Campinas. Faculdade de Enfermagem. Departamento de Saúde Coletiva. Campinas, SP, Brasil

**Keywords:** Premature Birth, Health Status Disparities, Indigenous Peoples, Brazil, Cross-Sectional Studies

## Abstract

**OBJECTIVE:**

To examine racial inequalities in preterm birth (PTB; < 37 weeks) among White, *Parda*, Black, and Indigenous mothers in Brazil’s Central-West region using a population-based cross-sectional design.

**METHODS:**

We analyzed 2,156,674 live births registered in *Sistema de Informação sobre Nascidos Vivos* (2015–2024) across Goiás, Mato Grosso, Mato Grosso do Sul, and the Federal District. Logistic regression with progressive covariate adjustment estimated odds ratios, with White mothers as the reference group; prevalence ratios from Poisson regression with robust variance, adjusted probabilities by group, and absolute risk differences were estimated as complementary measures.

**RESULTS:**

Crude preterm birth prevalence was 11.1% (White), 11.0% (*Parda*), 11.7% (Black), and 17.6% (Indigenous). In the primary sociodemographic model, Indigenous mothers had 50.4% higher odds (OR = 1.504; 95% CI 1.461–1.549), corresponding to a 28.9% attenuation of the crude OR. The adjusted probability of preterm birth was 15.9% among Indigenous *versus* 11.2% among White mothers (absolute difference 4.7 percentage points; prevalence ratio 1.41). In exploratory models additionally adjusting for prenatal care, parity, and marital status (n = 2,146,853), the Indigenous OR was attenuated to 1.222 (95% CI 1.186–1.259), although adjustment for prenatal visits is susceptible to reverse causation. Black mothers showed a modest crude excess (OR = 1.053) that did not persist after adjustment (full model OR = 0.973), and *Parda* mothers showed slightly lower odds throughout.

**CONCLUSION:**

Indigenous mothers in the Central-West experience a substantial preterm birth inequality that is only partially attenuated by measured sociodemographic factors and is compatible with the influence of unmeasured structural and contextual determinants.

## INTRODUCTION

In Brazil, preterm birth (PTB)—defined as delivery before 37 completed weeks of gestation—affects approximately 11% of all live births, accounting for over 340,000 premature neonates annually and representing the leading cause of neonatal mortality in the country^1,2^. Despite near-universal coverage of prenatal care and hospital delivery within the Unified Health System (SUS), PTB prevalence has remained stable over the past decade, with marked variation across regions and sociodemographic groups^3,4^. Racial health inequities in Brazil are rooted in centuries of slavery, colonialism, and the structural organization of society along racial lines^5,6^. Structural racism—the system through which racial hierarchies produce and reproduce unequal access to resources, political power, and health services—operates as a fundamental determinant of health for Black and Indigenous populations within the SUS^6,7^.

Black and Indigenous women bear a disproportionate burden of adverse perinatal outcomes, though through distinct pathways. Leal et al.^8^, in the nationwide Birth in Brazil study, documented that Black women received lower quality prenatal and obstetric care than White women even after propensity score matching, with *Parda* (mixed-race) women occupying an intermediate position. A meta-analysis estimated PTB risk to be approximately 50% higher among Black compared with White women^9^. Notably, most Brazilian perinatal studies aggregate Black and *Parda* into a single category, potentially masking heterogeneous risk profiles^8^. Indigenous populations face a qualitatively different configuration of disadvantage. Brazil is home to 1.69 million Indigenous people belonging to 305 ethnic groups, yet population-based evidence on their perinatal health remains scarce^12^. Available studies report elevated rates of infant mortality, low birth weight, and chronic undernutrition among Indigenous children^13^. A recent nationwide cohort of over 21 million newborns documented the highest attributable fractions of adverse birth outcomes among Indigenous women; however, its national-level analysis did not disaggregate findings by region^11^.

Brazil’s Central-West region is particularly suited to examine these inequalities. The region harbors one of the highest concentrations of Indigenous territories outside the Legal Amazon, home to peoples including the Xavante, Bororo, Terena, and Guarani-Kaiowá^12,16^. Simultaneously, rapid agricultural expansion and internal migration have produced a diverse, multiracial population, enabling direct comparisons across four racial groups—White, *Parda*, Black, and Indigenous—within a shared geographic and healthcare context. To date, no population-based study has simultaneously examined PTB inequalities across these four groups in the Central-West using a progressive adjustment framework.

This study aimed to: (1) estimate the association between maternal race/color and PTB among White, Brown, Black, and Indigenous mothers in Brazil’s Central-West region (2015–2024); (2) quantify the degree to which these associations are attenuated by sociodemographic and clinical factors through progressive covariate adjustment; and (3) characterize the residual association that persists after adjustment, which may be compatible with unmeasured structural determinants.

## METHODS

### Study Design and Data Source

This population-based cross-sectional study used data from the *Sistema de Informação sobre Nascidos Vivos* (SINASC – Brazilian Live Birth Information System), maintained by the Ministry of Health. SINASC compiles individual-level records from the Declaration of Live Birth (DN), completed at delivery in all Brazilian healthcare facilities, with coverage exceeding 95% of live births since 2011^17^. Data were accessed through the *Departamento de Informação e Informática do Sistema Único de Saúde* (DATASUS) public portal in Parquet format. Records for 2015–2023 corresponded to the consolidated national files; the 2024 file corresponded to the final consolidated DNBR release published by DATASUS on December 23, 2025, and accessed on June 27, 2026.

### Study Population

The source population comprised all live births registered in the Central-West region – Goiás, Mato Grosso, Mato Grosso do Sul, and the Federal District – between January 1, 2015, and December 31, 2024 (n = 2,338,990). Sequential exclusion criteria were applied: (1) missing, unknown, or Yellow/Asian maternal race/color (n = 159,207); (2) missing or biologically implausible gestational age (< 22 or > 44 weeks; n = 13,596); (3) missing maternal education (n = 9,482); and (4) implausible maternal age (< 10 or > 60 years; n = 31). The primary analytical sample comprised 2,156,674 live births (92.2% of the source). Because prenatal care, parity, and marital status are proximal variables used only in exploratory models, they were not required for the primary sample; the sensitivity subsample with complete data on these variables comprised 2,146,853 births (99.5% of the primary sample). Sequential exclusion counts are reported in Figure 1.

**Figure 1:**
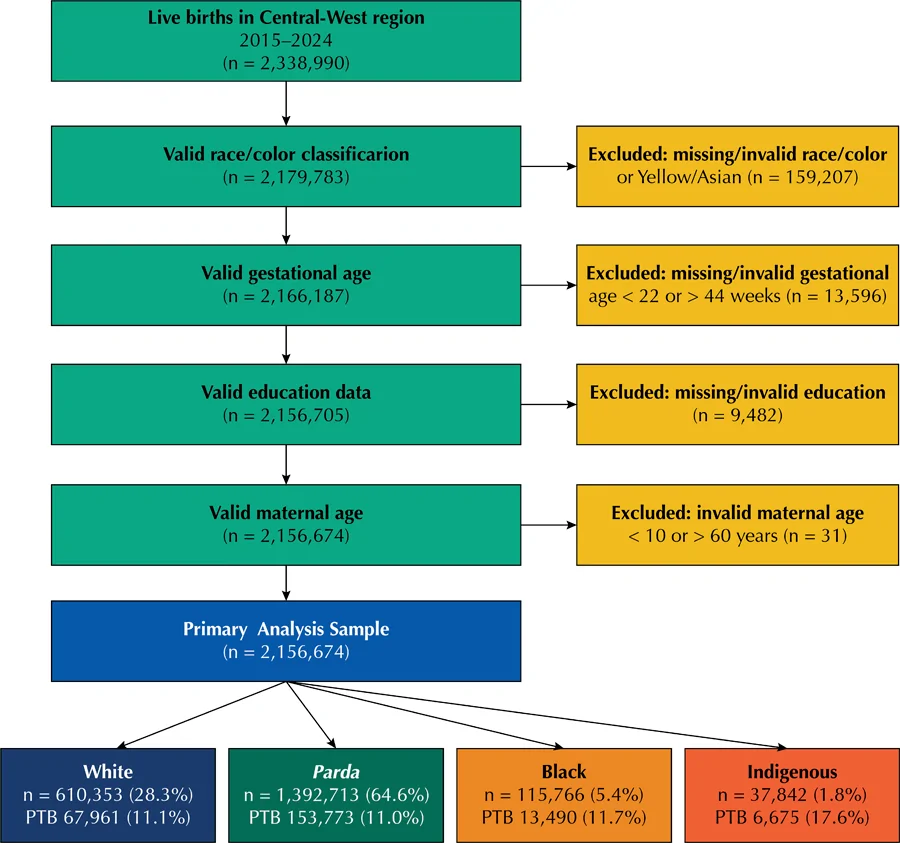
Flowchart of study population selection. Central-West region, Brazil, 2015–2024.

PTB: preterm birth.

Note: Sensitivity analysis subsample: n = 2,146,853 (99.5% of primary). Additional exclusions: missiong prenatal care, parity, or marital status (n = 9,821).

Because the largest single loss in the original specification stemmed from missing values in proximal covariates, we examined whether exclusions could have introduced selection bias by comparing included and excluded records. Indigenous mothers represented 1.8% of included births and a similar share (approximately 1.2%) of all excluded records, indicating that exclusions did not over-represent the Indigenous group; missing race/color was instead concentrated in the Federal District and Goiás and in earlier years. However, PTB prevalence was higher among excluded than included records (18.2% *versus* 11.2% among records with valid race/color), indicating that exclusions were not random with respect to the outcome. We therefore treated the complete-case analysis as the primary approach and interpreted the findings in light of this non-random exclusion, which we acknowledge as a limitation. Multiple imputation was not applied, because most exclusions stemmed from missingness in the exposure and outcome variables themselves, for which imputation offers limited advantage over complete-case analysis^18^.

### Outcome

PTB was defined as delivery before 37 completed weeks of gestational age, based on the SEMAGESTAC variable in SINASC, which records gestational age in completed weeks as estimated by the attending clinician.

### Exposure

The primary exposure was maternal race/color (RACACORMAE), classified into four groups: White (code 1, reference), *Parda* (code 4), Black (code 2), and Indigenous (code 5). Black and Parma were analyzed separately to enable detection of group-specific patterns^8^. Race/color in SINASC is recorded by the healthcare professional completing the DN, which may not always reflect maternal self-identification – a potential source of misclassification, particularly for Indigenous women in non-Indigenous facilities. We did not assume a single direction for this bias: misclassification could attenuate the observed Indigenous–White inequality if Indigenous women are recorded as non-Indigenous, but could also operate in the opposite direction in specific settings; we therefore interpret estimates cautiously rather than as necessarily conservative^19^.

### Covariates

Covariates were organized into a hierarchical framework following Victora et al.^20^, in which distal sociodemographic determinants are entered before proximal healthcare factors. For the primary models (Models 1–5): maternal age (< 20, 20–34 [reference], ≥ 35 years); educational attainment (five SINASC ordinal levels, entered as categorical indicators); birth year (2015–2024, categorical); and state of residence (Goiás, Mato Grosso, Mato Grosso do Sul, Distrito Federal, categorical). For the exploratory models (Models 6–8), three additional covariates were incorporated: prenatal care visits (four SINASC categories, entered as categorical indicators); previous live births (parity); and marital status (with/without partner).

Conceptual framework. Our primary analytical target was the total inequality in PTB between racial groups (Models 1–2) and its attenuation after adjustment for distal sociodemographic factors (Models 3–5), which we interpret as confounders or contextual descriptors of social position rather than as biological exposures^21^. Proximal factors entered in Models 6–8—prenatal care, parity, and marital status—lie on the causal pathway between social position and PTB and may partly constitute the phenomenon under study; adjusting for them can therefore remove part of the inequality itself. We accordingly treat Models 6–8 as exploratory and do not interpret the corresponding attenuation as a causal decomposition^22^.

### Statistical Analysis

Associations between race/color and PTB were estimated using multivariable logistic regression with progressive adjustment. Eight sequential models were fitted: Model 1 (crude); Model 2 (+age); Model 3 (+education); Model 4 (+birth year); Model 5 (+state — primary model); Model 6 (+prenatal care); Model 7 (+parity); Model 8 (+marital status — full model). Odds ratios (OR) with 95% confidence intervals were the primary measure. The proportion by which each adjusted OR moved toward the null was reported as the percent attenuation of the OR, computed as [(OR_crude − OR_adjusted)/(OR_crude − 1)] × 100 only when the crude OR exceeded 1.0; this descriptive quantity does not represent a formal causal decomposition. Because PTB is not a rare outcome, we additionally reported prevalence ratios from Poisson regression with robust variance^23^, absolute risk differences, and model-based adjusted probabilities by group as complementary measures. To account for clustering of births within municipalities, standard errors were clustered by municipality of residence (CODMUNRES). Given the very large sample, interpretation emphasized the magnitude and epidemiological relevance of associations rather than statistical significance alone. Model fit was assessed by Akaike Information Criterion.

Heterogeneity was examined by estimating PTB prevalence by race and state, testing a race×state interaction by likelihood-ratio test, and conducting analyses stratified by Mato Grosso do Sul and Mato Grosso; temporal variation was assessed by year and by pre-pandemic, pandemic, and post-pandemic blocks. The manuscript was revised in accordance with the STROBE checklist for cross-sectional studies.

### Sensitivity Analyses

Prespecified analyses evaluated robustness: (1) restriction to singleton births; (2) exclusion of 2024 to assess the influence of the most recent year; (3) an alternative educational classification (ESCMAE2010) to assess dependence on the schooling variable; (4) prenatal care operationalized as initiation in the first trimester (a measure less dependent on gestational duration) rather than the total number of visits. The comparison of included and excluded records (Study Population) further assessed potential selection bias. All analyses used a fixed random seed.

### Software

All analyses were performed in Python 3.13 using statsmodels 0.14.6, pandas, and NumPy (random seed 42)^a^.

### Ethical Considerations

This study used exclusively secondary, anonymized, and publicly available data from SINASC/DATASUS. It was approved by the Universidade Estadual de Campinas Research Ethics Committee (Of. CEP no. 036/2026); ethics review is waived under Brazilian National Health Council Resolution 510/2016^21^.

## RESULTS

*Parda* mothers constituted most of the analytical sample (n = 1,392,713; 64.6%), followed by White (n = 610,353; 28.3%), Black (n = 115,766; 5.4%), and Indigenous (n = 37,842; 1.8%) (Table 1). Indigenous mothers were markedly younger, with substantially lower educational attainment, and were geographically concentrated on Mato Grosso do Sul and Mato Grosso. In the sensitivity subsample, only 62.9% of Indigenous mothers initiated prenatal care in the first trimester, compared with 88.8% of White mothers.

**Table 1:** Maternal and birth characteristics by racial group, Central-West region, Brazil, 2015–2024 (Base B; n = 2,156,674).

Characteristic	White	Parda	Black	Indigenous
Total births, n	610,353	1,392,713	115,766	37,842
Preterm birth				
Preterm birth, n (%)	67,961 (11.1)	153,773 (11.0)	13,490 (11.7)	6,675 (17.6)
Extreme (< 28 wk), %	0.4	0.5	0.6	0.5
Very (28–31 wk), %	0.9	0.9	1.1	1.5
Moderate/late (32–36 wk), %	9.8	9.6	10.0	15.6
Maternal age				
Mean (SD), years	28.4 (6.5)	26.4 (6.5)	27.2 (6.6)	23.9 (6.8)
< 20 years, %	9.7	15.7	12.7	30.9
20–34 years, %	70.6	71.3	71.9	60.4
≥ 35 years, %	19.7	13.1	15.4	8.7
Educational attainment				
None, %	0.1	0.2	0.3	8.0
1–3 years, %	0.5	1.1	1.3	8.9
4–7 years, %	5.6	12.5	12.5	31.6
8–11 years, %	45.7	65.9	65.1	46.8
≥ 12 years, %	48.2	20.5	20.8	4.7
State of residence				
Goiás, %	37.3	42.3	40.4	2.7
Mato Grosso, %	22.1	26.5	28.9	40.1
Mato Grosso do Sul, %	24.2	16.8	10.9	55.8
Federal District, %	16.4	14.4	19.7	1.5
Prenatal care visits (sensitivity subsample)				
None, %	0.9	1.1	1.4	6.6
1–3 visits, %	3.5	5.9	6.7	12.8
4–6 visits, %	15.8	22.3	22.6	36.0
≥ 7 visits, %	79.6	70.4	68.9	44.2
Unknown, %	0.3	0.3	0.4	0.5

SD: standard deviation; wk: week.

Note: primary analysis sample (n = 2,156,674), updated to the reproduced Base B; values are column percentages unless otherwise indicated. Prenatal care is shown for the sensitivity subsample (n = 2,146,853). First-trimester prenatal initiation (among records with a recorded month) was 88.8% (White), 83.2% (Parda), 81.5% (Black), and 62.9% (Indigenous); the month of initiation was missing for 2.3%, 3.2%, 3.6%, and 10.7%, respectively. Following reviewer guidance, p-values were omitted in favor of measures of magnitude.

A total of 241,899 PTB occurred, an overall prevalence of 11.2%. Indigenous mothers had the highest rate (17.6%), followed by Black (11.7%), White (11.1%), and *Parda* (11.0%); the absolute Indigenous–White difference was 6.5 percentage points (Table 1).

In unadjusted analysis (Model 1), Indigenous mothers had 70.9% higher odds of PTB (OR = 1.709; 95%CI 1.663–1.757), corresponding to a crude prevalence ratio of 1.58 (Table 2; Figure 2). Black mothers showed a modest excess (OR = 1.053), while *Parda* mothers exhibited marginally lower odds (OR = 0.991). For Indigenous mothers, age adjustment changed the OR only slightly (Model 2: 1.699), education produced the largest reduction (Model 3: 1.586), and adjustment for birth year (Model 4: 1.559) and state (Model 5: OR = 1.504; 95%CI 1.461–1.549; 28.9% attenuation of the crude OR) yielded further, diminishing attenuation. In the primary model, the adjusted probability of PTB was 15.9% among Indigenous *versus* 11.2% among White mothers (absolute difference 4.7 percentage points), and the adjusted prevalence ratio was 1.41 ( 95%CI 1.38–1.45). Approximately 70% of the crude excess persisted after sociodemographic adjustment.

**Table 2:** Progressive adjustment models for racial inequalities in preterm birth, Central-West region, Brazil, 2015–2024.

Model	n	Parda OR (95%CI)	Black OR (95%CI)	Indigenous OR (95%CI)	Attenuation %
M1 Crude	2,156,674	0.991 (0.981–1.000)	1.053 (1.032–1.074)	1.709 (1.663–1.757)	-
M2 + Age	2,156,674	1.000 (0.990–1.010)	1.061 (1.040–1.082)	1.699 (1.652–1.746)	1.4
M3 + Education	2,156,674	0.985 (0.975–0.995)	1.044 (1.024–1.065)	1.586 (1.541–1.633)	17.3
M4 + Year	2,156,674	0.981 (0.971–0.991)	1.026 (1.005–1.046)	1.559 (1.514–1.605)	21.2
M5+ State [PRIMARY]	2,156,674	0.989 (0.979–0.999)	1.035 (1.014–1.056)	1.504 (1.461–1.549)	28.9
M6 + Prenatal	2,146,853	0.944 (0.934–0.954)	0.965 (0.946–0.985)	1.214 (1.178–1.251)	69.8
M7 + Parity	2,146,853	0.950 (0.940–0.959)	0.969 (0.950–0.989)	1.224 (1.188–1.261)	68.4
M8 + Marital [FULL]	2,146,853	0.952 (0.943–0.962)	0.973 (0.954–0.993)	1.222 (1.186–1.259)	68.7

OR: odds ratio; 95%CI: 95% confidence interval.

Note: prevalence ratios (Poisson, robust variance, clustered by municipality), Indigenous: M1 = 1.58 (1.55–1.62); M5 = 1.41 (1.38–1.45); M8 = 1.17 (1.15–1.20). Percent attenuation reported only for Indigenous (crude OR > 1). Models 6–8 are exploratory. % attenuation of the OR = [(OR_crude − OR_adjusted)/(OR_crude − 1)] × 100.

Reference: White mothers. Odds ratios (primary) and prevalence ratios (complementary).

**Figure 2:**
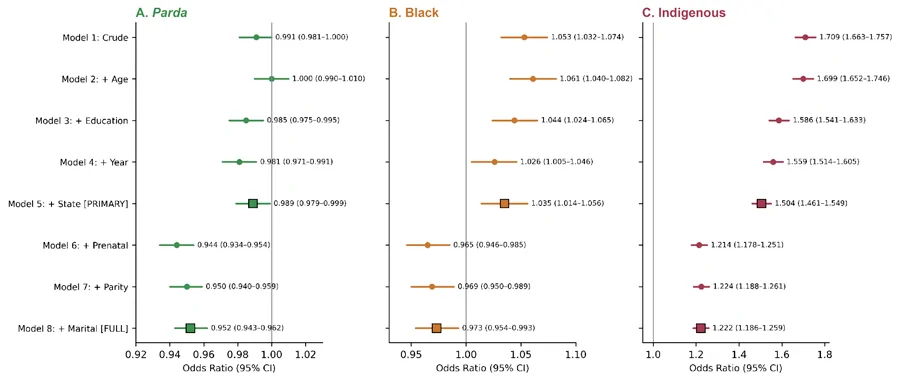
Crude and adjusted odds ratios for preterm birth by maternal race/color.

Note: White reference. Squares mark the primary (Model 5) and full (Model 8) models.

In the exploratory models (Models 6–8), adjustment for prenatal care produced the largest single shift in the Indigenous OR, reducing it to 1.214 (Model 6); parity and marital status produced little further change (full model, Model 8: OR = 1.222; 95%CI 1.186–1.259; prevalence ratio 1.17, 95%CI 1.15–1.20). Because shorter gestations afford less time to accumulate prenatal visits, this attenuation may partly reflect reverse causation rather than a causal pathway, and these models are interpreted as exploratory. For Black mothers, the modest crude excess did not persist after adjustment, with the full-model OR crossing the null (Model 8: 0.973; 95%CI 0.954–0.993). *Parda* mothers showed slightly lower odds throughout (Model 8: 0.952; 95%CI 0.943–0.962).

Heterogeneity by state and period. PTB prevalence among Indigenous mothers varied markedly across states, reaching 22.0% in Mato Grosso do Sul *versus* 12.0–12.8% in the other units, and a race×state interaction was supported (likelihood-ratio χ^2^ = 697.3; df = 9; p < 0.0001). In the primary model, the Indigenous association was strong in Mato Grosso do Sul (OR = 1.883; 95%CI 1.811–1.957) but near null in Mato Grosso (OR = 1.014; 95%CI 0.958–1.073). After full adjustment, the Mato Grosso estimate fell below 1.0 (OR = 0.750), an implausible “protective” value that we interpret as an illustration of the over-adjustment expected when conditioning on prenatal care, consistent with the exploratory status of Models 6–8. The Indigenous association was stable across pre-pandemic, pandemic, and post-pandemic periods (OR ≈ 1.50 in all three blocks).

Prenatal care measurement. When prenatal care was operationalized as initiation in the first trimester rather than the number of visits – a measure less dependent on gestational duration – the Indigenous association remained higher (OR = 1.540) than when adjusting for the number of visits (OR = 1.337), consistent with reverse causation affecting the visit-count specification.

Estimates were consistent under the alternative educational classification (ESCMAE2010: Model 5 OR = 1.560; Model 8 OR = 1.301), exclusion of 2024 (Model 5 OR = 1.590; Model 8 OR = 1.320), and restriction to singleton births, in which the Indigenous association was slightly stronger (Model 5 OR = 1.586; Model 8 OR = 1.271; n = 2,109,640), indicating that retaining multiple gestations in the primary analysis was conservative.

## DISCUSSION

This population-based study of more than 2.1 million live births in the Central-West region (2015–2024) revealed a pronounced and Indigenous-specific PTB inequality. Indigenous mothers had 70.9% higher crude odds (OR = 1.709), of which approximately 70% persisted after sociodemographic adjustment (Model 5: OR = 1.504). Black mothers showed a modest crude excess that did not persist after adjustment, and *Parda* mothers exhibited lower odds than White mothers across all models. These findings challenge the assumption of a generalized racial gradient and instead point to determinants specific to the Indigenous experience.

This inequality must be interpreted through the lens of structural racism – institutional arrangements and historical processes that systematically produce disadvantage for racialized groups^25^. For Indigenous peoples in Brazil, structural racism is inseparable from territorial dispossession, forced displacement, and exclusion from state institutions^28,29^. The residual association is compatible with the influence of unmeasured upstream factors – land insecurity, environmental degradation, disruption of food systems, and the cumulative physiological burden of discrimination – consistent with the weathering hypothesis^30^; these factors were not measured in this study and are advanced as hypotheses rather than demonstrated mechanisms.

The magnitude of this disparity mirrors patterns across settler-colonial societies, where Indigenous women experience elevated PTB relative to non-Indigenous women in Australia, Canada, and New Zealand, confirmed as a structural feature by the Lancet–Lowitja Global Collaboration^31^.

Differences in prenatal care were associated with the largest attenuation of the Indigenous association in exploratory models. This is consistent with marked gaps in care access: only 62.9% of Indigenous mothers initiated prenatal care in the first trimester, compared with 88.8% of White mothers. Geographic barriers are substantial, as Indigenous births are concentrated in Mato Grosso do Sul and Mato Grosso, where the *Subsistema de Atenção à Saúde Indígena* (SasiSUS) faces persistent challenges^12,13,16,35^. Nationally, the adequacy of prenatal care is strongly patterned by maternal sociodemographic characteristics^38^. However, because adjustment for the number of prenatal visits may embed reverse causation and over-adjustment, we refrain from describing prenatal care as the main mediating pathway and emphasize that the residual Indigenous association (OR = 1.222) indicates that measured factors do not fully account for the inequality.

The marked heterogeneity across states—with a strong association in Mato Grosso do Sul and a near-null crude association in Mato Grosso—indicates that a regional average obscures distinct state-level patterns, supporting the reviewer’s call for a stratified analysis. The stability of the Indigenous association across pre-pandemic, pandemic, and post-pandemic periods is notable given the disproportionate impact of Covid-19 on Indigenous peoples in Brazil^39^.

This study’s strengths include substantial statistical power, the four-group classification, and robustness across alternative specifications. Several limitations should be acknowledged. The cross-sectional design precludes causal inference. Race/color misclassification, particularly for Indigenous women in non-Indigenous facilities, may bias estimates in either direction. Exclusions were not random with respect to the outcome (PTB prevalence was higher among excluded records), a potential source of selection bias that we assessed by comparing included and excluded records and acknowledge as a limitation. SINASC does not capture important mediators—including tobacco use, nutritional status, household income, and gestational diabetes—nor distinguish spontaneous from provider-initiated PTB. Finally, findings are specific to the Central-West and may not generalize to other regions.

Strengthening prenatal care within SasiSUS is essential, and Indigenous-led maternity models such as Birthing on Country offer promising frameworks^40^. As a substantial part of the inequality remained after adjustment, intersectoral policies encompassing land regularization, food sovereignty, and environmental protection are equally necessary^13,29^. In conclusion, Indigenous mothers in the Central-West face a distinct PTB burden that is only partially explained by measured factors and is compatible with structural determinants requiring both targeted healthcare expansion and accountability for the colonial legacies that shape Indigenous women’s health.

## Footnotes

a Analysis code is available from: https://github.com/guico2lh-dot/ind-ptb-CO/tree/v1.0
